# Targeting Feeding and Eating Behaviors: Development of the Feeding Dynamic Intervention for Caregivers of 2- to 5-Year-Old Children

**DOI:** 10.1155/2015/964249

**Published:** 2015-06-23

**Authors:** Ihuoma U. Eneli, Tracy L. Tylka, Rosanna P. Watowicz, Jessica Hummel, Jan Ritter, Julie C. Lumeng

**Affiliations:** ^1^Center for Healthy Weight and Nutrition, Nationwide Children's Hospital, 700 Children's Drive, Columbus, OH 43205, USA; ^2^Department of Pediatrics, Ohio State University, Nationwide Children's Hospital, 700 Children's Drive, Columbus, OH 43205, USA; ^3^Department of Psychology, Ohio State University, 225 Psychology Building, Columbus, OH 43210, USA; ^4^Ohio Action for Healthy Kids, 370 South Fifth Street, Columbus, OH 43215, USA; ^5^Center for Human Growth and Development, University of Michigan, 300 North Ingalls Street, Ann Arbor, MI 48109, USA; ^6^Department of Pediatrics, University of Michigan, 1500 E. Medical Center Drive, Ann Arbor, MI 48109, USA; ^7^Human Nutrition Program, Department of Environmental Health Sciences, University of Michigan School of Public Health, 1415 Washington Heights, 1700 SPH I, Ann Arbor, MI 48109, USA

## Abstract

Targeting feeding dynamics, a concept centered on the roles and interaction of the caregiver and child in a feeding relationship, may have significant potential for obesity intervention. The aim of this paper is to describe the 3-phase development of the Feeding Dynamics Intervention (FDI), an acceptability and feasibility study on implementing the feeding dynamic roles (Study 1), development of the FDI content (Study 2), and a pilot study on use of the 6-lesson FDI to promote behaviors consistent with a feeding dynamic approach (Study 3). Sample population was mothers with young children, 2–5 years old. An effect size (Hedges' *g*) greater than 0.20 was seen in more than half (57%) of maternal feeding behaviors, with the largest effect sizes (Hedges' *g* ≥ 0.8) occurring with behaviors that represent the mother adopting her roles of determining what food is served, not using food as a reward, and not controlling her child's intake. There was a significant decline in Pressure to Eat behaviors (2.9 versus 2.2, *p* < 0.01) and Monitoring (4.1 versus 3.5, *p* < 0.001). The FDI emerged as an acceptable and implementable intervention. Future studies need to investigate effects of the FDI on the child's eating behaviors, self-regulation of energy intake, and anthropometrics.

## 1. Introduction

The 2007 American Medical Association Expert Committee on Prevention, Assessment, and Treatment of Childhood Obesity identified caregiver feeding behaviors as an important target for intervention, categorically stating that there is consistent evidence to support “avoiding overly restrictive feeding behaviors” [1]. A growing body of the literature indicating that caregiver feeding behaviors, in particular overly restrictive or controlling behaviors, significantly reduce a child's ability to self-regulate their energy intake [2–4] and result in weight gain or a higher body mass index [4–6] shaped the expert committee's guidelines. One area related to caregiver feeding behaviors is feeding dynamics, which is centered on the roles, interactions, and balance of control between the caregiver and child in a feeding relationship.

Satter described an ideal feeding dynamics environment as one in which the caregiver decides “what” food is offered, “where” food is eaten, and “when” food is eaten and allows the child to decide “whether to eat” as well as “what” and “how much” to eat of the food offered [7, 8]. She originally proposed this feeding dynamics approach as the “trust model.” Satter posits that children learn to trust and develop a healthy relationship with food when they and their caregivers each have an appropriate level of control and autonomy in the feeding relationship. Interventions invoking these concepts may be effective as an obesity prevention or treatment strategy [9]. The primary premise of feeding dynamics is based on nurturing optimal self-regulation of energy intake to support normal growth. Young children have an innate ability to self-regulate their energy intake in response to their metabolic needs [10–12]. This natural aptitude weakens as children get older and become more responsive to external influences on eating [13–15]. Children with the highest body fat and whose parents restrict their food choices and quantity of intake exhibit the weakest level of regulation [16].

However, this relationship is complex, not always consistent, and probably bidirectional; that is, the child's characteristics, for example, age, gender, and weight, influence caregiver eating or feeding practices and vice versa [4, 17–19]. For instance, mothers who eat regular meals and choose nutritious foods for themselves are more likely to extend these behaviors to their children [20]. Mothers who eat intuitively (giving themselves unconditional permission to eat, eating for physical rather than emotional reasons, and relying on their internal hunger and satiety cues to guide eating) restrict their children's food intake less and allow their children to share feeding responsibilities [20]. The child's perception of the caregiver's controlling behavior can contribute to the complexity of the relationship [21, 22]. Limiting the amount and types of snack foods available in the home, a covert controlling action, is associated with decreased intake of unhealthy snacks [22, 23], while feeding practices, like pressuring the child to eat, are associated with an aversion to healthy food choices and a higher risk of overweight [4, 24]. The challenge is to determine the right balance of control between the caregiver and the child.

Despite the complexity of the relationship, a review of the literature supported the feeding dynamic approach as a viable paradigm for obesity prevention or treatment and is described in depth elsewhere [15]. What remains uncertain is how best to teach mothers to adopt these feeding roles, whether mothers will be willing or able to implement the roles, and how these roles can be adapted in an obesogenic environment, as scholars have not yet developed or tested this approach as a comprehensive program. The goal of this paper is to describe the development of the Feeding Dynamics Intervention (FDI), a parent-targeted program based on the feeding dynamics approach in addition to evidence-based lifestyle behaviors known to prevent or treat childhood obesity. We developed the FDI using three independent projects. The hospital institutional review board approved each of these projects.

## 2. Study 1: Acceptability and Implementation of the Components of Feeding Dynamics

In Study 1, we tested the acceptability and implementation of the feeding dynamic components.

Specifically, we initially piloted a 90-minute class to determine how best to teach the feeding dynamic roles and to obtain feedback from the mothers of 2–5-year-old children regarding their perception and experience implementing the feeding roles.

### 2.1. Method

#### 2.1.1. Participants and Procedure

We asked mothers of young children from two childcare centers if they would be willing to attend a 90-minute learning session with other mothers about how to feed their children. A total of 22 mothers indicated that they would like to attend a learning session. Participants completed a survey prior to and 4–6 weeks following the class. We invited mothers, as opposed to fathers, because mothers are typically the parent responsible for grocery shopping and meal preparation [7]. Of these 22 mothers, 17 attended the class and fourteen mothers completed both the pre- and postsurveys. They implemented the recommendations for a mean of 22 days (range 10–42). Mothers' mean age was 32.8 (SD = 4.2) years, and their children's mean age was 3.6 (SD = 1.2) years. They were identified as White (50%) and African American (50%). As their highest level of education, they had graduated from high school (47%) or from a 4-year college (50%); 3% did not graduate from high school.

#### 2.1.2. Measures

We assessed feeding behaviors using the Child Feeding Questionnaire (CFQ) [25] and the Caregiver Feeding Responsibility Scale (CFRS) [26]. For the CFQ Restriction (8 items), Monitoring (3 items), and Pressure to Eat (4 items) subscales, higher scores reflect greater levels of the measured feeding behavior. Restriction ranged from 1 (*disagree*) to 5 (*agree*), and Monitoring and Pressure to Eat ranged from 1 (*never*) to 5 (*always*). Much psychometric evidence supports the CFQ. In the current study, Cronbach alphas were 0.73 for Restriction, 0.73 for Pressure to Eat, and 0.82 for Monitoring. We used the CFQ parent perception of child's weight subscale to determine weight status. Responses are markedly underweight, underweight, normal weight, overweight, and markedly overweight.

The CFRS measures the feeding dynamic roles for caregivers and children as proposed by Satter [7]. Specifically, items ask mothers about the extent to which they perform their responsibilities (e.g., feeding their children at regular times, serving meals with a variety of foods, and ensuring that the family eats together without distractions such as TV) and allow their children to perform their responsibilities (i.e., deciding what or how much to eat of what has been offered). With a response scale ranging from 1 (*never*) to 5 (*always*), higher scores indicate greater adherence to the feeding dynamic approach [26]. The CFRS factor structure is unidimensional, demonstrates internal consistency reliability (*α* = 0.70), test-retest reliability over a 5-week period (*r* = 0.80), and constructs validity among mothers of 2- to 5-year-old children [26].

Postsurvey measures include the CFQ and CFRS. We also developed six independent questions on mothers' prior familiarity with, as well as understanding and acceptability of, the feeding dynamic approach (scale ranging from 1 (*strongly disagree*) to 5 (*strongly agree*)). Specifically, two of these six questions queried mothers on the extent to which they knew about and fed their children according to principles within the feeding dynamic approach* before* taking the class—if they were more familiar with the feeding dynamic approach prior to the class, then they would have less to gain from the class. One question asked whether they understood the feeding dynamic approach after the class to determine whether it was delivered in a clear and informative manner. The remaining three questions asked about their acceptability of the feeding dynamic approach, that is, the extent mothers believed that this approach would be beneficial for children, started feeding their children according to this approach, and planned to continue feeding their children according to this approach. Higher scores indicate greater familiarity, understanding, and acceptability.

#### 2.1.3. Feeding Dynamics Class

The class content, adapted from Ellyn Satter's work on caregiver/child division of feeding responsibility, focused on how to (a) implement each of the caregiver roles (i.e., “what” food is offered, “where” food is eaten, and “when” food is eaten) and (b) allow and support the child in carrying out his/her roles (“whether” to eat, “what” and “how much” to eat of the food offered) [7, 8]. The first author taught the classes. She defined each role, emphasized the benefits of carrying out and supporting the roles, and provided step-by-step guidance on implementation. For example, in presenting the caregiver role of “where” food is eaten, the instructor recommended the caregiver designate an area in the home where all food and drink can be consumed except water. The instructor presented the benefit of undertaking such an action first from a caregiver perspective (e.g., you will have a cleaner home) and then from the child's perspective (e.g., if you maintain this rule in your home, your child will learn to associate eating with specific areas in the home at an early age and limit the likelihood he/she will eat in front of the TV or bedroom). Next, the instructor discussed how to implement the role of “where.” She advised caregivers to have a family discussion about the action (e.g., limiting where food and drinks are consumed to a designated area) a couple of days before the change and present the change as an opportunity for the family to encourage each other for the shared benefit of a cleaner home. She recommended that the caregiver and family select a designated area in the home for food or drinks (preferably the kitchen).

The instructor elicited input and problem-solved challenges and barriers raised by the participants, such as an uncooperative spouse or family, how to identify and prepare the physical space for the meal, or the absence of a kitchen or dining table in the home. She taught participants to share any potential exceptions to the rule ahead of implementation (e.g., we can eat in the living room but only on family fun nights). She advised them to try to implement each recommendation at least 80% of the time during the 4–6-week trial period. Participants provided informal feedback on each recommendation for a significant proportion of the class time.

#### 2.1.4. Data Analysis

We used paired *t*-tests to assess change in feeding behavior, between responses on the pre- and postintervention surveys. We applied the Bonferroni adjustment to avoid a type I error due to multiple comparisons. Because five items/scales on the maternal feeding behaviors from pre- to postintervention were skewed and/or kurtotic (defined as skewness/standard error > |2|), we transformed these variables using reflect and square root transformations for four variables that were moderately negatively skewed and square root transformations for one variable that was moderately positively skewed [27]. These transformations reduced skewness and kurtosis to acceptable levels, so paired-sample *t*-tests could be performed. For ease of interpreting means, the nontransformed means are presented. Effect size (Hedges' *g*) for each feeding behavior determined the magnitude in change following the class. We used Hedges' *g* in lieu of Cohen's *d* to estimate effect size because Hedges' *g* provides a better estimate for small sample sizes [28]. However, the effect sizes are comparable in interpretation. Specifically, per Cohen [29], we considered an effect size of 0.20 a small effect, 0.50 a medium effect, and 0.80 a large effect. We conducted all analyses with SPSS 19.0.

### 2.2. Results

Based on the independent questions developed by the research team (range 1–5 with higher scores indicating greater familiarity, acceptability, and experience), mothers did not feed their children according to the feeding dynamic approach (*M* = 2.00, SD = 0.7) and indicated that they were not familiar with the approach (*M* = 2.00, SD = 0.9) prior to the intervention. After intervention, mothers reported that they understood the feeding dynamic approach (*M* = 4.50, SD = 0.6), believed that children should be fed according to the feeding dynamic approach (*M* = 4.22, SD = 0.6), planned to make lasting changes in how they feed their children (*M* = 4.29, SD = 0.5), and were somewhat able to implement the changes (*M* = 3.45, SD = 1.0).

Changes in maternal feeding behaviors from pre- to postintervention are shown in Table 1. The nontransformed means are presented in Table 1. We noted that an effect size (Hedges' *g*) greater than 0.20 appeared in more than half (57%) of maternal feeding behaviors (Table 1), with the largest effect sizes (Hedges' *g* ≥ 0.8) occurring with behaviors that represent the mother adopting her responsibility for determining what food is served, not using food as a reward, and not controlling her child's intake. The mothers' lowest scores were related to allowing children to carry out their eating roles. Based on the CFQ subscales, controlling behaviors such as Restriction, Pressure to Eat, and Monitoring decreased significantly from pre- to postintervention (Table 1).

At postintervention, mothers provided feedback on areas they thought would be challenging to implement or needed clarification. They recommended a guide on how much food to prepare or serve, without appearing to waste food. They anticipated that it would be difficult not to control or restrict their children's food intake, and they had a preference to plate their children's food at certain meals. Mothers reported that they needed information on nutrition label reading, were insistent that label reading would help them be more knowledgeable when purchasing food, and verbalized that label reading should be a key component of any nutrition intervention. They identified commonly anticipated barriers, which included how to implement the recommendations with older children and nonsupportive family members, how to share the approach with extended family members who may also care for the child, and how to talk with their children about hunger and satiety. Despite the small sample size, there was an improvement in behaviors aligning with the feeding dynamic approach following the class (Table 1).

## 3. Study 2: Developing and Refining the FDI Program

Guided by our prior work in this area [9, 20, 30, 31] and results from Study 1, a research team of two dietitians, a physician, a curriculum expert, and psychologist developed the 6-lesson FDI. We designed Study 2 to evaluate the FDI program content in three evaluations over six months and finalize its content.

### 3.1. Method

#### 3.1.1. Participants and Procedure

An evaluation team of three dietitians, five health educators, and a public health specialist from the university extension program in eight rural and urban Ohio counties conducted the initial program evaluation. Age of evaluators ranged from 33 to 56 years, and all were Caucasian, had taught families for a minimum of five years, and currently worked with low-socioeconomic clientele of different ethnicities. In this initial evaluation, we assessed the evaluators' perceptions about the feeding dynamic approach, the likelihood of their clients adopting these roles, and how best to present the material and identify benefits and challenges. They evaluated each lesson for content, ease of teaching, applicability for families, and appropriateness of supporting materials. The evaluators spent one day with the research team reviewing the FDI framework and content. They completed written and verbal feedback on each lesson and provided feedback during discussions with the research team.

Subsequently, a group of five reviewers, which included parents and two of the initial evaluators, conducted the second and third program evaluations. The parents were volunteers from one of the childcare centers. Their ages ranged from 26 to 35 years, two had children who were toddlers and preschoolers, and one parent had a child who was overweight. In these second and third evaluations, we refined the FDI content based on results from the literature reviews, the pilot study (Study 1), feedback from the evaluators, and research on feeding dynamics [9, 20]. We structured each session to last 3-4 hours, not including time spent reviewing the material prior to the meeting.

### 3.2. Results

The evaluators reported all the caregiver and child roles in the feeding dynamic approach would be applicable for their clients. Two evaluators expressed concern that allowing children to determine how much to eat would be challenging for parents who have children with impaired satiation. However, they all reported they would feel comfortable teaching the FDI content after only a brief training session.

The final FDI content included information that (a) caregivers are responsible for offering nutritious foods and exposing children to new foods (the “what”), providing structured snack and mealtimes to decrease indiscriminate snacking or grazing (the “when”), identifying designated areas for eating or drinking within the home (the “where”), and sitting and eating with children and keeping the eating atmosphere pleasant (family meals and role modeling) and (b) children are responsible for what to eat and how much (or even whether) to eat from the food provided (Table 2). We also included evidence-based lifestyle messages, for example, reducing sweetened beverages, having family meals, serving balanced meals, decreasing fast food consumption and eating out, reducing TV viewing, and increasing physical activity in the FDI [1, 32].

In the FDI, we discouraged tactics such as drinking water before meals, waiting 20 minutes between servings, or putting down the fork between bites for the sole purpose of food restriction. We also discouraged offering effusive praise like clapping hands, making smiley faces, or offering rewards for eating, as these behaviors constitute Pressure to Eat and may promote food consumption in the absence of hunger. Instead, we incorporated techniques on how to use neutral phrases, such as “you must have enjoyed your macaroni and cheese tonight,” rather than “wow, that's too much macaroni and cheese” in the FDI curriculum. At each lesson, we reviewed the continuum from hunger to fullness to feeling “stuffed” and taught mothers to communicate these concepts to their young children. These changes are essential as they may improve the mother's self-efficacy in carrying out the recommendations and strengthen her perception that the FDI will enable her to feed her child well and help her child learn to eat well.

In the final FDI curriculum, we added a module to help mothers recognize their own hunger and satiety cues and be mindful of their eating behavior to each lesson in response to results from a prior study [20] that found mothers who had positive eating behaviors (i.e., those who ate intuitively) were more likely to allow their children to carry out their own feeding roles. This addition provides an opportunity for role modeling, a social-cognitive theory (SCT) construct used in the development of the FDI. We also incorporated feedback obtained from the 90-minute class (Study 1). To address mothers' concern about how much food to prepare or serve without appearing to waste food and control or restrict their children's intake, we encouraged mothers to use at least 1.5 portions per child when cooking as a guide. After much debate, the team decided to allow mothers to plate some meals as family style meals may not be realistic for certain meals/settings and could have a varied effect on portion size and energy intake in some children. For plating, the FDI instructs mothers to provide approximately three-fourths of a portion size initially, allowing the child to have additional servings if requested. We used these guidelines to provide a consistent approach within the curriculum and facilitate mothers' adherence. Because mothers in Study 1 were unanimous that nutrition label reading should be a key component of any nutrition intervention, we included a brief section in the fourth lesson (Table 3). We presented nutrition label reading as a guide to help with balancing food choices through the day or week, rather than restricting intake or food choices. In response to their feedback about anticipated barriers and the diversity in feeding behaviors, we included case scenarios in the lessons to allow for problem solving.

Recommendations from the reviewers included incorporating more hands-on activities, use of video clips to demonstrate division of responsibility roles, strengthening the authoritative parenting component of the sessions, emphasizing issues around food security, and using more pictures in the handouts. There was significant debate about the use of video clips drawn from contemporary television shows to illustrate feeding and parenting styles, as some reviewers felt it would limit the use of the curriculum in resource-poor settings. The team decided to include these video clips as they demonstrated strong visual examples of the feeding roles. In addition, they felt that, with rapid technological advancement, this concern may be less relevant in the future even in resource-poor areas. With each review, we identified and incorporated learner objectives and processes in structuring the content (Table 3). With these modifications, the FDI content deviated in significant ways from the trust model as proposed by Satter [7, 8].

## 4. Study 3: Pilot Feasibility Study of the FDI

In the final study, we piloted the final curriculum of FDI to assess whether mothers who received the intervention adopted the caregiver roles and decreased excessive controlling feeding behavior (i.e., Restriction and Pressure to Eat).

### 4.1. Method

#### 4.1.1. Participants and Procedure

We piloted the comprehensive 6-lesson FDI over 12 weeks with a convenience sample (*N* = 8) of mothers recruited through an email sent to a parent-teacher association for the local school district. We targeted this parent-teacher association because the mothers in this association had children in the targeted age range for the FDI, the school was situated in the same county as the childcare centers, and mothers lived near the facility where the FDI would take place to ensure that distance was not a deterrent to completing the FDI. A registered dietitian taught the FDI. Seven mothers completed the intervention. The eighth mother withdrew prior to the start of the intervention due to family reasons. At each lesson, mothers set goals for home and provided feedback on the content in a pre- and posttest survey. We set aside time for group discussions on their experience implementing their goals. Characteristics of the sample population are described in Table 4. We noted that sample characteristics in Studies 1 and 3 are comparable in terms of child age, although mothers in Study 3 were slightly younger and less educated than mothers in Study 1.

#### 4.1.2. Measures

The participants completed a survey prior to and following the classes that included items on demographics, maternal height and weight, the CFQ, and the CFRS. The postsurvey included similar measures and the six questions on perception, acceptability, and experience with feeding dynamic approach.

#### 4.1.3. Data Analysis

We used paired-sample *t*-tests to compare differences between responses on the pre- and postintervention surveys.

### 4.2. Results

Mothers reported that they were not familiar with the feeding dynamic approach before the intervention (*M* = 1.71, SD = 0.7 on a scale ranging from 1 =* strongly disagree* to 5 =* strongly agree*). After the intervention, they agreed that children should be fed according to the FDI (*M* = 4.00, SD = 1.0), strongly agreed that they understood the feeding dynamic approach (*M* = 4.57, SD = 0.5), and indicated a willingness (*M* = 4.14, SD = 1.46) and a plan (*M* = 4.43, SD = 0.8) to continue to feed their children according to the FDI. Mothers felt neutral with regard to whether it was difficult to feed their children according to the FDI (*M* = 2.86, SD = 1.2) and strongly agreed that the FDI should be used with all children, regardless of if they are overweight (*M* = 4.43, SD = 0.5), thin or underweight (*M* = 4.43, SD = 0.5), or average weight (*M* = 4.43, SD = 0.5). Therefore, these mothers' data show that they viewed the FDI as both acceptable and fairly feasible. Following the intervention, six of the seven mothers who completed the intervention reported less restrictive and pressuring practices on the CFQ (Table 4). Concern over their children's weight decreased following the intervention (preintervention: *M* = 3.2, SD = 1.4; postintervention *M* = 2.3, SD = 1.2).

Six of the seven mothers reported that they were able to implement the following strategies: making one meal for all family members (instead of “short order cooking”), letting children choose what to eat from what is on the table, having children drink only water between meals and snacks, and having food off-limits between meals and snack times. Five of the seven mothers reported that they were able to let the children decide how much they want to eat from what was served and when they were done with eating. For the two participants that indicated otherwise, one had not yet tried and the other did not want to try it. Participant #3 disagreed with the roles in a feeding dynamic relationship, especially the child's roles. She was Latino, normal weight, married, and college educated and had an 11-month-old child in addition to the 5-year-old child in the study. She lived with her mother who was also Latino. She expressed concerns about cultural appropriateness of some of the recommendations and shared it was rude for the child not to taste from what had been served. She strongly believed in the importance of parental control over all feeding decisions. In summary, all but one participant responded well to the 6-lesson FDI; mothers were able to implement the recommendations, and the level of controlling feeding behaviors improved.

## 5. Discussion

We used the results gleaned from these studies to further refine the program by including (a) a section on how feeding behaviors may be perceived by the child, (b) a discussion on the intersection between FDI recommendations and cultural beliefs, (c) a review of parental and child feeding roles in each lesson to reemphasize the core concepts of feeding dynamics (we used this review to obtain periodic feedback on participants' progress and to encourage mothers who have not yet tried the recommendations to do so), (d) a 3-dimensional child-friendly “stomach model” that illustrates three stomachs in different states (i.e., hungry, full, and stuffed) and a module to instruct mothers how to use the stomach model with their young children, (e) team exercises on meal planning using paper food models, and (f) physical activity resource handouts on relevant community physical activity resources and an indoor play activity sheet. The activity sheet consists of 4-5 aerobic activities such as running in place, which the child and family can perform in 30–60-second intervals. The objectives of the FDI in delineating child and caregiver feeding responsibilities and incorporating* what* children are fed within the broader framework of* how* they are fed enable caregivers to achieve the overall goal of feeding their children well, an act that evokes strong emotions tied to a sense of accomplishment as a parent.

This paper makes several new contributions to the literature. First, we developed the FDI, an innovative program which incorporates evidence-based recommendations for childhood obesity and a core focus on feeding dynamics. From our studies, we modified and expanded on the feeding dynamic concepts by adapting them appropriately to be pragmatic, cognizant of the prevailing obesogenic environment, and responsive to the mother's needs and diversity in the child's eating behaviors and patterns. Second, we uncovered that mothers were receptive to the feeding dynamic approach concepts, found them understandable, and were willing and able to implement the intervention at home. Third, the FDI decreased controlling and restrictive feeding behaviors. The rigorous stepwise development of the studies allowed us to incorporate a wide range of data, prior research, and participant feedback which strengthened the final FDI; however, limitations exist. The most significant limitation is the small sample size within each study. We used convenience samples to pilot test the intervention; thus, the mothers in our samples may have been more receptive to an intervention than the general population. We used the newly developed Caregiver Feeding Responsibility Scale [26] to assess the feeding dynamic components, which needs further validation in different populations. Additional limitations include report bias because the study relied solely on maternal reports and some of the items and subscales had relatively large standard deviations, which could impact effect size levels. The FDI needs to be carried out in larger samples before definitive conclusions are made.

Finally, based on the feedback from the mothers, the evolving research on feeding and eating behaviors in young children, and our research, the final FDI program contains significant deviations from the trust model [7, 8] These changes include the addition of a module on portion sizes to guide the amount of food to cook, lack of insistence on family style meals, and introducing label reading to guide selections of nutritious options. The addition of evidence-based lifestyle recommendations beyond feeding behaviors further strengthened the FDI, increasing its potential to be a promising and practical program for obesity prevention or treatment in young children. There are many next steps in this area of research [33]. They include researchers (a) investigating the outcome of the FDI with a larger and diverse population, (b) studying the degree to which children can be taught to recognize hunger and satiety cues via parent-directed intervention versus a child-targeted intervention, (c) videotaping within the home to assess the feeding environment rather than relying on caregiver self-report, (d) using objective assessments of self-regulation such as the energy compensation and eating in the absence of hunger tests, (e) validating the acceptability and feasibility of the physical activity component, and (f) exploring short- and long-term effects of the FDI on the child's anthropometrics and eating behaviors.

## Figures and Tables

**Table 1:** Maternal feeding behaviors before and after the 90-minute class (Study 1).

Item	Preintervention mean (SD)	Postintervention mean (SD)	*p* value	Hedges' *g *
*I feed my child at regular times instead of waiting until he/she asks for food*.	3.86 (0.9)	4.14 (0.6)	0.263	0.36
*I allow my child to eat as much as she/he wants to eat from what is offered*.	4.00 (1.0)	3.86 (1.1)	0.635	0.13
*I allow my child to stop eating when she/he seems full*.	4.29 (0.7)	4.21 (0.6)	0.775	0.12
*My child eats in front of the T.V. or computer*.^a^	1.86 (0.9)	1.71 (0.9)	0.547	0.16
I use food as a reward.^a,b^	3.46 (1.0)	1.92 (1.0)	0.002	1.50
*We have pleasant conversations during meals that include everyone*.	3.86 (0.9)	3.82 (0.8)	0.893	0.05
*I only prepare food for my child that she/he likes to eat*.^a,b^	3.21 (0.8)	2.53 (0.4)	0.004	1.04
*My child eats wherever she/he wants to eat in the house*.^a^	2.36 (1.0)	1.93 (1.0)	0.111	0.42
*We eat meals together as a family*.	3.86 (0.7)	3.71 (0.7)	0.547	0.21
I allow my child to have drinks other than water between meals.^a^	3.36 (1.0)	3.21 (1.0)	0.710	0.15
I allow my child to have other food whenever she/he doesn't like the meal.^a,b^	3.07 (0.9)	2.00 (0.6)	0.006	1.36
My child gets her/his own food when hungry.^a^	2.38 (1.0)	2.00 (0.7)	0.240	0.43
*I serve meals with a variety of different foods*.	4.16 (0.6)	4.21 (0.6)	0.672	0.08
I try to make my child eat everything on her/his plate.^a,b^	3.43 (1.0)	2.14 (1.1)	0.030	1.19
Caregiver Feeding Responsibility Scale (CFRS)				
Total score (from italicized items above)	3.82 (0.52)	3.98 (0.44)	0.099	0.32
Child Feeding Questionnaire subscales				
Restriction^b^	3.51 (0.7)	3.25 (0.6)	0.000	0.39
Pressure to Eat	2.93 (1.0)	2.26 (0.7)	0.011	0.76
Monitoring	4.11 (0.8)	3.57 (0.8)	0.006	0.66
Concern about child's weight	3.31 (1.4)	2.89 (1.2)	0.332	0.31

*Note*. Italics indicate Caregiver Feeding Responsibility Scale (CFRS) items. CFRS item response scale: 1 = never, 2 = rarely, 3 = sometimes, 4 = often, 5 = always. Postsurvey was completed 4–6 weeks following intervention.

^a^These items were reverse-scored before the total CFRS score was calculated. For these items, lower Time 2 scores are expected.

^b^Denoting statistical significance *p* < 0.00263 (Bonferroni adjustment; 0.05/19 = 0.00263).

**Table 2:** Topics in the Feeding Dynamic Intervention (Study 2).

Session	Topic
1	Parent's role in feeding and child's role in eating

2	Limiting where and when food is eaten—“the where and when”

3	Parenting styles: family meals/balanced meals—“the what, when, and where”

4	Nutrition —“the what”: practice meal and menu planning

5	Problem-solve “the what” scenarios and introduce physical activity

6	Physical activity and wrap-up

**Table 3:** The Feeding Dynamic Intervention learner objectives and processes (Study 2).

Learner objectives	Process incorporation in FDI
Concrete experience	Using or referring to participants' prior experiences to engage participants in a new experience. For example, in Lesson 1, mothers reflect on their experiences of eating as children and current experiences with eating and feeding their children.

Reflective observations	Reflecting on prior or current experiences to help take action steps. For example, in Lessons 4 and 5, we use their current practices to teach how to take action steps to improve picky eating or refusal of new foods.

Abstract conceptualization	Allowing participants to identify relevancies, general truths and form conclusions specific to their needs upon which to act. For example, in Lessons 3 and 4, mothers will learn to use the balanced plate concept and practice to relate to general recommendations for servings of food groups.

Active experimentation	Guiding and coaching participants to implement or experiment with new behaviors. For example, in Lesson 2, mothers are taught a stepwise approach to implement limiting where food is eaten in the home to a designated area. In Lesson 5, a cooking demonstration is performed.

**Table 4:** Outcomes of the Feasibility of Feeding Dynamic Intervention (Study 3).

Characteristics						^*∗*^CFRS		^*∗∗*^CFQ Restriction		^*∗∗*^CFQ pressure		^*∗∗*^CFQ concerns	
Subject	Maternal age (yrs)	Edu.	Ethn.	Maternal weight	Child's Age (yrs)	Before	After	Before	After	Before	After	Before	After
1	29	HS	Blk	Obese	5	3.7	4.3	4.29	1.71	1.25	1.00	4.67	1.00
2	26	HS	Cauc.	Normal	3	4.0	4.1	3.29	3.29	3.75	2.5	3.33	2.67
3	26	COL	Cauc.	Normal	5	4.3	3.5	3.29	4.57	2.00	3.25	4.00	3.33
4	27	GRA	Cauc.	Normal	4	4.4	4.6	3.86	3.29	3.5	1.5	1.00	3.67
5	26	HS	Cauc.	Obese	2	4.0	4.4	3.43	2.86	1.00	1.00	3.33	3.00
6	22	HS	Latino	Overweight	3	3.3	4.2	4.14	3.14	4.25	3.25	4.33	1.67
7	21	HS	Blk	Obese	3	3.8	4.2	3.43	2.57	2.00	2.00	1.67	1.00

^*∗*^Caregiver Feeding Responsibility Scale (CFRS); higher scores indicate more of the identified characteristics.

^*∗∗*^Child Feeding Questionnaire (CFQ); higher scores indicate more of the identified characteristics.

Edu.: education; HS: high school graduate; COL: college graduate; GRA: postgraduate degree; ethn.: ethnicity; Cauc.: Caucasian; Blk: Black.

BMI ≥ 30 (obese); BMI 25–29.9 (overweight); BMI 18.5–24.9 (normal).
